# The Self-Adaptation Ability of Zinc Oxide Nanoparticles Enables Reliable Cancer Treatments

**DOI:** 10.3390/nano10020269

**Published:** 2020-02-05

**Authors:** Zane Taylor, Marcelo Marucho

**Affiliations:** 1 Department of Applied Physics and Materials Science, California Institute of Technology, Pasadena, CA 91125, USA; zwtaylor76@gmail.com; 2 Department of Physics and Astronomy, University of Texas at San Antonio, One UTSA Circle, San Antonio, TX 78249, USA

**Keywords:** zinc oxide nanoparticles, electrical double layer, smart anti-cancer agents, cancer therapy, nanomedicine

## Abstract

Optimal procedures for reliable anti-cancer treatments involve the systematic delivery of zinc oxide nanoparticles, which spread through the circulatory system. The success of these procedures may largely depend on the NPs’ ability of self-adapting their physicochemical properties to overcome the different challenges facing at each stage on its way to the interior of a cancerous cell. In this article, we combine a multiscale approach, a unique nanoparticle model, and available experimental data to characterize the behavior of zinc oxide nanoparticles under different vessels rheology, pH levels, and biological environments. We investigate their ability to prevent aggregation, allow prolonged circulation time in the bloodstream, avoid clearance, conduct themselves through the capillarity system to reach damaged tissues, and selectively approach to target cancerous cells. Our results show that non-functionalized spherical zinc oxide nanoparticles with surface density *N* = 5.89 × 10^−6^ mol/m^2^, protonation and deprotonation rates *pKa* = 10.9 and *pKb* = −5.5, and NP size in the range of 20–50 nm are the most effective, smart anti-cancer agents for biomedical treatments.

## 1. Introduction

**Figure 1:**
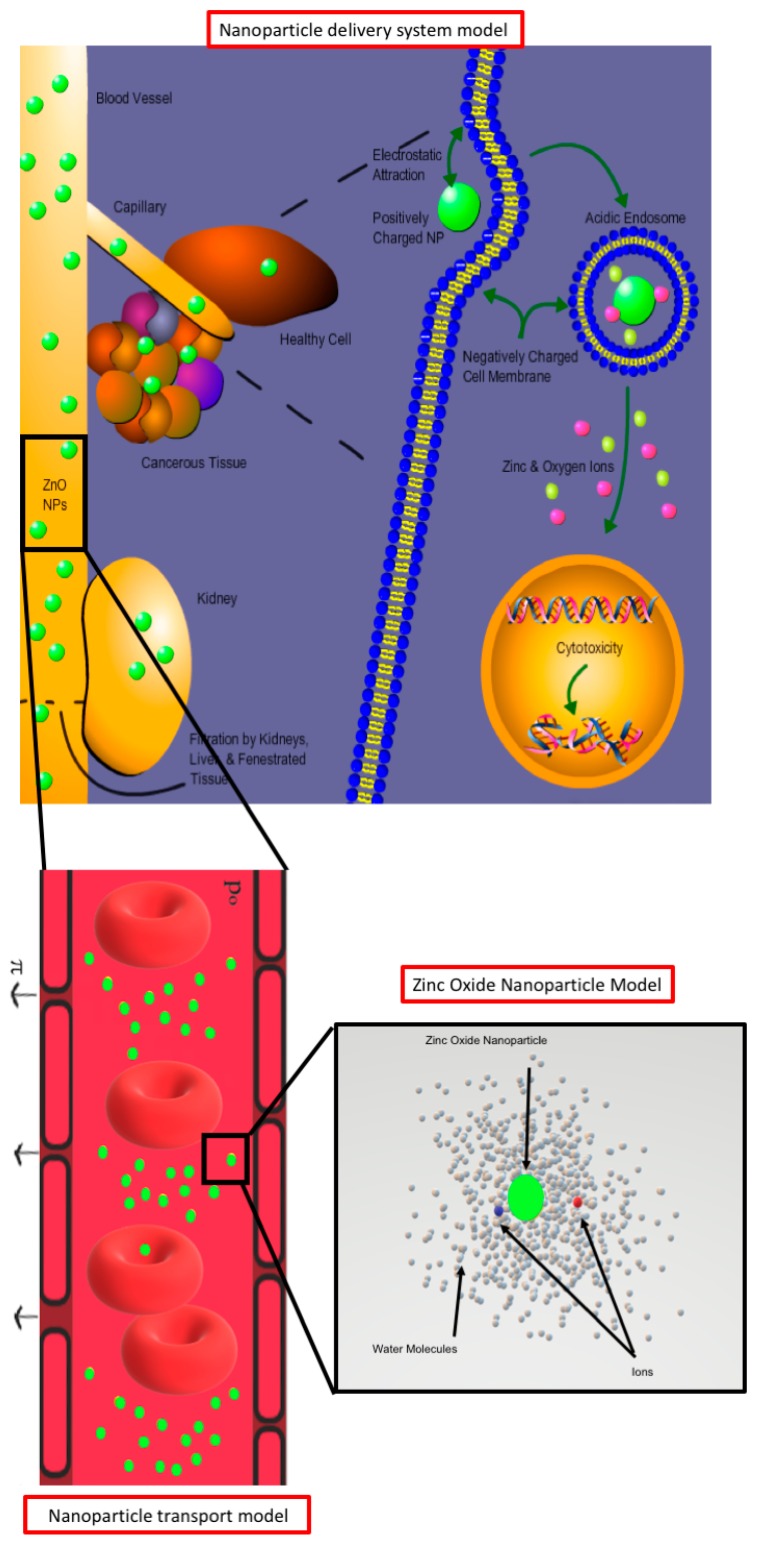
Multi-scale approach.

While conventional therapies face the significant drawback of targeting and killing almost as many healthy cells as cancerous ones, others lack vectors (or transporting vehicles such as nanoparticles) adequately capable of bypassing the numerous tumorous and biological barriers [1]. Among the substantial number of in-vitro studies on metallic oxide NPs as anticancer agents, zinc oxide (ZnO) nanoparticles (NPs) are of particular interest. Their relatively high biocompatibility at non-nanoscale sizes for long-circulation time treatments [2], the high solubility at low pH [3,4,5], the creation of cytotoxic reactive-oxygen-species (ROS) in the presence of ZnO NPs [6,7,8,9,10,11], in conjunction with the enhance permeation and retention (EPR) effect noted within cancerous tissues [2], demonstrate their high potential for anticancer agents. If systemically delivered in human beings, these NPs must survive passage through the circulatory system (Figure 1).

This approach might be possible if ZnO NPs are able to modify their physicochemical properties in response to its environment to pass through each stage of the treatment efficaciously. Without functionalization, the most predominant characteristic of NPs is the nanoparticle size (NS). Other relevant physicochemical properties of NPs, such as zeta potential (ZP) and surface charge density (SCD), are also subject to changes due to their strong dependence on specific environmental conditions and NP surface parameters such as the number of chemical reactive sites on the NP surface and its protonation and deprotonation constants.

These parameters play a crucial role in electrical NP interactions. ZP represents the electric potential difference between the stationary layer of electrolyte attached to the surface of the NP and the bulk solution. Whereas, SCD measures the molar surface density of charge upon the NP [12]. When systematically delivered in the circulatory system (see Figure 1), these physicochemical properties may exhibit contrasting behavioral prerogatives in many ways:

- ZP large enough in magnitude to prevent NP aggregation [13], but small enough not to provoke rapid clearance into all neighboring tissues [2,14,15].
- NS small enough not to be filtered out by the liver and kidney, [1,15] but not so small as to be absorbed by all tissues the circulatory system may reach [1,16,17].
- NS large enough to appropriately conduct NPs through the microvascular flow with little effective diffusion towards smaller vessels to prevent rapid clearance to healthy tissues [18] and promote longer-term dosages.
- Balanced interplay between NS, ZP, and SCD that generate strong long-range Coulombic forces, dampened by the salts in biological fluids, to pull NPs out of the circulatory system and through the interstitium, against contrary convection, to the vicinity of the cancerous cells themselves [19].
- NS producing enough binding energy to pull NPs through the cancerous membrane with either endocytosis or phagocytosis [1,7,15,20,21,22,23].
- ZP large enough to induce high cytotoxicity through reactive-oxygen-species (ROS) and dissolution [6,7,8,9,10,11].

Thus, a comprehensive study on the suitability of a systematic delivery system for ZnO NPs must include every treatment stage to account for all these challenges [24]. In this article, we combine innovative approaches and models, as well as, available experimental data to investigate the ZnO NPs ability of self-adapting their physicochemical properties to overcome the different challenges facing at each stage on its way to the interior of a cancerous cell. We use a recently introduced classical solvation density functional theory (CSDFT) combined with a new complexation surface (titration) model for ZnO NPs to characterize the impact of biological environments on their physicochemical properties [25,26,27]. CSDFT has been shown to be in a good agreement with numerical simulations and experimental results in a variety of benchmark systems including silica oxide nanoparticles under multiple electrolyte conditions. This formalism accounts for the rearrangement of water molecules and ions surrounding the NP, which form an electrical double layer (EDL) around the NP surface (see Figure 1). It also accounts for the impact of the pH on the SCD arising from the protonation/deprotonation reactions of the functional groups actively lying on the NP surface.

**Figure 2:**
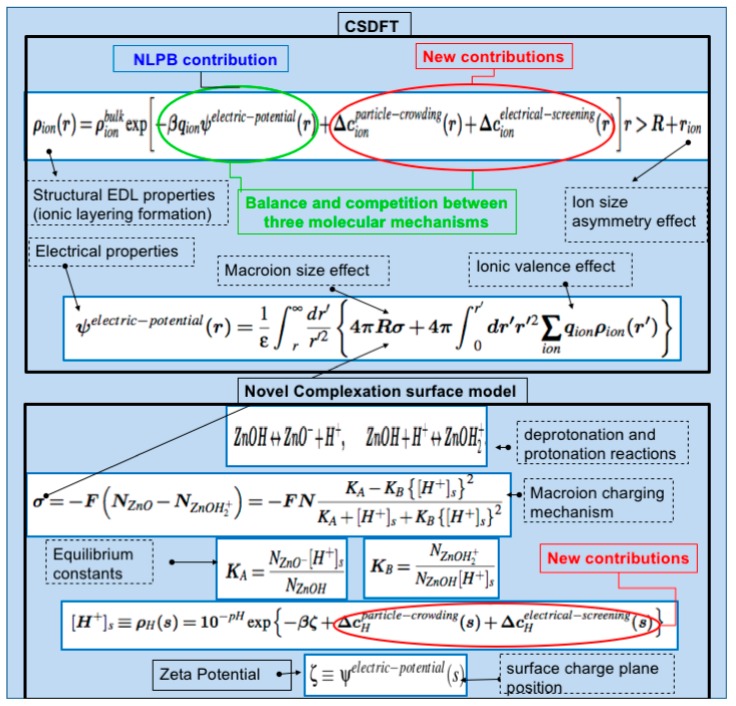
Fundamental equations to calculate surface charge density and zeta potential for ZnO NPs. Parameters and expressions for the new contributions can be found in references 26 and 27. Numerical implementation of the approach is provided in the freely available software CSDFTS [29].

The primary advantage of this approach over conventional solvation theories for NPs [28] is the inclusion of not only the electrostatic but also the steric and inter-ionic charge interactions (see red color in Figure 2). These two latter interactions, usually omitted in simplistic approaches, are necessary to account for the particle crowding and the inter-ionic electrostatic charge screening arising from the ion-ion coulombic force in the ion cloud surrounding the NP. The particle crowding plays a key role in NP hydration and the ionic layering formation, whereas the inter-ionic electrostatic charge screening mainly contributes to the effective NP charge and the ZP. By taking these ingredients into account properly and efficiently, the approach provides a more realistic characterization of ZnO NPs under a variety of aqueous salt solutions. We perform numerical calculations using the open source software CSDFTS [29] to estimate the impact of pH level, biological environment condition (bulk ion concentrations and species), and NS on the ZP and SCD, as well as the excess number of free Zn and O ions surrounding the NP. This software comes with a graphical user interface which allows users without programming and computational skills to perform such calculations. Additionally, the unique model for a single ZnO NP described above, the Casson fluid model (for the plasma and red blood cells), and a suitable transport theory for NPs in circulatory systems [18] are combined in this work to describe the impact of NS, vessel permeability and blood rheology on the effective diffusion of ZnO NPs in large vessels (arteries), capillary, and venules. We also use the Derjaguin, Landau, Vervey, and Overbeek (DLVO) theory [19,30], the approach proposed for a single ZnO NP, and an spherical model for rigid charged cells [31,32] to estimate the relative tendency of ZnO NP deposition onto healthy and cancerous cell membranes as well as the NP stability under a variety of biological environments, pH level and NS. Moreover, assuming that the electrostatic interaction is the dominant long-range force between particles in a biological system, we use Ohshima et al.’s approach [33], the models for a single ZnO NP and a cell mentioned above to investigate the electrostatic selectivity mechanisms involved in NP-Cell interactions for different NP sizes and electrolyte conditions. We calculate the ratio between different NP-Cell systems to determine the impact of ionic strength of the biological fluid, NS, and ZP on the selectivity approach. (further information on these approaches and numerical calculations is provided in the Supplementary Material for reproducibility purposes).

## 2. Materials and Methods

### 2.1. A Unique Complexation Surface Model for ZnO NPs

As a first approximation, we represent the NP as a rigid charged sphere of various radius R (=NS). The SCD is characterized by the site density number *N* of active oxide functional groups *ZnO* uniformly lying on the NP surface. Each active functional group can protonate (absorb a positive electron charge, *ZnO*^+^) or deprotonate (release a positive electron charge, *ZnO*^−^). The protonation and deprotonation rates are given by the intrinsic constants *pKa* and *pKb*, respectively. The number of functional groups protonated and deprotonated depends on the surface parameters *N*, *pKa*, *pKb*, as well as the pH level, the biological environment condition, and the ZP of the NP, being that the difference between these two numbers is proportional to the *SCD* of the NP [25,26,27]. Using previous notation, [26,27] this unique complexation surface model for zinc oxide NPs is fully characterized by the expressions provided in Figure 2.

The surface site density *N* may be estimated from the Pivovarov formula [34], which relates the lattice constants to surface site density (e.g., *N* = 5.89 × 10^−6^ mol/m^2^). Additionally, we determine the parameters *pKa* and *pKb* to reproduce experimental results on the ZP of ZnO NPs obtained from light scattering techniques [35,36]. The optimal values are found to be: *pKa* = −Log(*Ka*) *=* 10.9 and *pKb* = −Log(*Kb*) *=* −5.5. (See Supplementary Material for more information).

Using this model and the CSDFT predictions for the ion density distributions, we calculate the excess zinc and oxygen ions trapped or excluded by individual NP’s double layers [37]. Electrostatically excluded zinc ions may be associated with one of the most efficient cytotoxicity mechanisms of ZnO NPs for killing cancerous cells [6,38,39] (See Supplementary Material for more information).

### 2.2. Biological Environment Models

We consider NPs surrounded by a biological environment comprised of ionic species with ion sizes obtained from crystallographic (Shannon) experiments [40]. In considering the solvent model, we represent the water molecules as a neutral ion species at the experimental size (2.75 A) and concentration (55.54 M), whereas, the electrostatics is considered implicitly by using the continuum dielectric environment with a dielectric constant Epsilon = 78.5 (see Figure 1).

We provide in Table 1 the bulk ionic composition of the biological environments (blood plasma, interstitial fluid, and intracellular fluid). For the sake of computational cost, we accurately simplify these biological electrolytes to the most concentrated species. Charged amino acids and proteins are neglected since the CSDFT cannot accommodate them. Instead, oxygen ions are used to maintain the charge neutrality of the difference.

The pH of the biological environment is controlled by adding small concentrations of a strong base (NaOH) and acid (HCl) solution to the biological fluid.

### 2.3. ZnO NPs Circulatory Transport

The ability of NPs to target and enter tumor tissues from blood is highly dependent on their behavior under blood flow. While in large vessels, blood can be treated as a Newtonian fluid displaying laminar flow profiles, the nature of blood (consisting of plasma plus red blood cells) cannot be omitted in capillaries and venules (microcirculation).

In this article, we use the Casson fluid model described by Gentile et al. to characterize the transport properties of ZnO NPs under a variety of vessel permeability (*L_p_* and π_i_) and blood rheology (*R_c_* and *R_e_*) conditions (see Supplementary Material for more information) [18].

**Figure 3:**
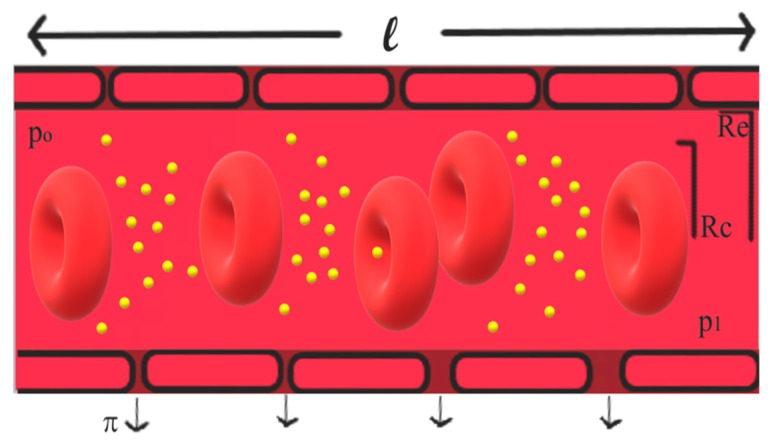
NP transport model.

The Casson fluid model not only describes well the blood nature but also provides efficient, analytical expressions to calculate the steady state longitudinal diffusion coefficient *D_eff_*. The blood flow is represented by a variable concentration of red blood cells, which tend to accumulate in the central core region of the capillary represented by the radius *R_c_*. This model leaves a marginal cell-free layer with thickness *d* (equal to *R_e_* − *R_c_*, where *R_e_* is the capillary radius) for the NP to be transported (see Figure 3). Additionally, the approach accounts for blood viscosity, η, and hemotocrit, H (blood volume percentage of red blood cells), which reduce with the vessel diameter. While the capillary walls may be permeable to the fluid, they are usually impenetrable and not adsorbent to NPs. In this work, we consider the vessels and blood flow tabulated in Table 2, the hydraulic conductivity and permeability parameters described in Table 3, and the pressure drops given in Table 4. In our analysis on circulatory transport, we simulate capillary sizes of 10 and 70 microns in diameter, use an accurate approximation for the rheological parameter ξ and model NP dimensions using an approximate hydrodynamic radius (NS plus the Debye length of the blood plasma described in Table 1 [1]).

### 2.4. NP-NP and NP-Cell Models for Short-Range Interactions

Although particle deposition onto surfaces and particle aggregation is a complex process that involves many factors such as particle size, surface charge heterogeneity, surface roughness as well as steric and hydrophobic interactions, both the electrostatic and van der Waal interactions are expected to play a significant role in the process. Based on this premise, Derjaguin, Landau, Vervey, and Overbeek developed an accurate and efficient approach which estimates association energy rates *k* in terms of the electrostatic and van der Waal interactions arising within the electrical double layer [19,30]. We use the approach to describe NP-NP interactions and NP-cell interactions at short separation distances and, consequently, the impact of ZP, PS, pH level, and biological environment condition on the NP aggregation and NP adsorption onto cell membranes.

The approach requires the characterization of the NP and cell, as well as, the van der Waals (Hamaker constant) parameters. While we use the model for a single ZnO NP described above, the cell is represented as a charged rigid sphere of experimental radius *R* = 12.5 micrometers, and ZP = −94 mV for some cancerous cells and ZP = +94 mV for healthy cells [31,32]. The Hamaker constants are intrinsic parameters to any system of two materials separated by a third medium. We use the Hamaker constants for the water-vacuum-water, zinc oxide-vacuum-zinc oxide, and phospholipid bilayer-vacuum-phospholipid bilayer interactions to obtain approximate values for the Hamaker constants for the zinc oxide-water-phospholipid bilayer and zinc oxide-water-zinc oxide systems. The resulting values for this model are H = 2.01 × 10^−20^ J and H = 1.92 × 10^−20^ J, respectively. (see Supplementary Material for more details).

## 3. Results

### 3.1. Impact of the Biological Environment on the Behavior of ZnO NPs

**Figure 4:**
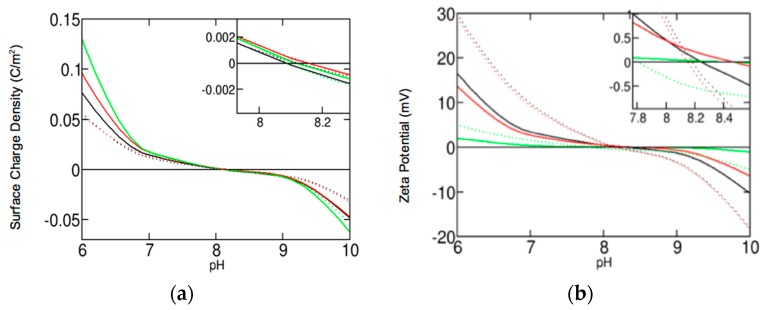
(**a**) Comparison of surface charge density as a function of pH for NS 4 nm (solid lines) and 250 nm (dotted lines) in blood plasma (black color), interstitial fluid (red color), and intracellular fluid (green color) described in Table 3. (**b**) Comparison of zeta potential as a function of pH.

The isoelectric point (IEP) and point-of-zero-charge (PZC) are found to be around pH 8.2 for any given condition (see Figure 4). ZP and SCD are positive (repulsive) for pH values below the IEP, with larger magnitudes displayed at lower pH. Whereas, they are negative (attractive) for pH values above the IEP, with larger magnitudes displayed at higher pH.

In agreement with available experimental data [43], the values predicted for ZPs and SCDs are slightly different for the blood plasma and interstitial fluid electrolytes regardless of the NS and pH in the biological condition. These differences vanish for NS larger than 250 nm. A different scenario occurs for intracellular fluid, which shows a significant decrease and increase in the ZP and SCD magnitudes, respectively. As NS increases, the ZP increases and the SCD decreases (see Figure 4). NPs displayed similar electrostatic behavior for biological fluids with and without dissolution (explored in the Supplementary Material). Near the IEP, these differences are negligible, and away from the IEP, they are less than 10%.

### 3.2. Blood Circulation

**Figure 5:**
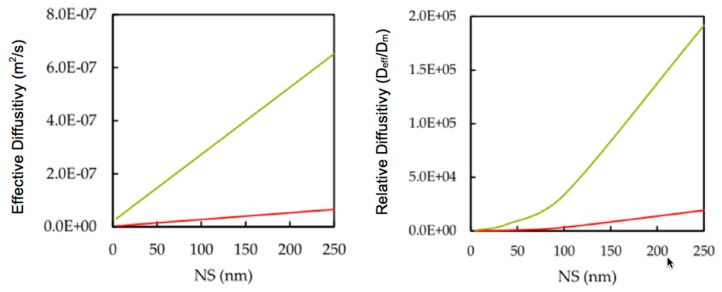
Effective and relative NP diffusivity of NPs with NS. 10 µm capillary size is given in red and 70 µm capillary size in green. The source tissue of the capillaries had no effect on their effective diffusivities.

We considered NS of 4, 40, 100, and 250 nm in radius and the parameters described in Table 2 to calculate the effective diffusion of ZnO NPs within the kidney, skeletal muscle, and tumorous capillaries of 10 and 70 microns in diameter. In agreement with previous work [18], we found a constant, effective diffusivity across all capillaries of the same size but in different tissues as shown in Figure 5. In all cases, the hydraulic properties of the capillary had no effect on the effective diffusion of the NP, reducing to a simple linear dependence on both the NS and the capillary size.

### 3.3. Long-Range Interactions

**Figure 6:**
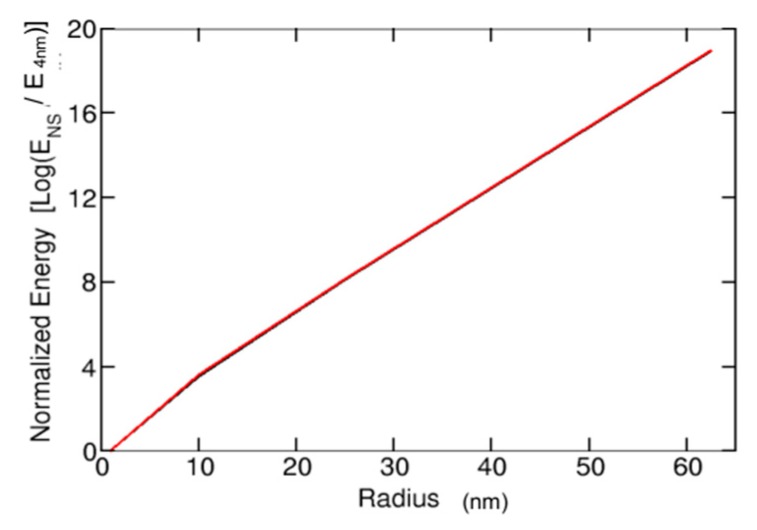
Comparison of the effects of NS on interaction energy with cells. Radius is normalized in terms of multiples of the base NS of 4 nm. The interaction energy is normalized in this way and presented in terms of the base-10 logarithm. Black line represents NPs in cancerous interstitial fluid (approx. pH 5), and red line represents NPs in healthy blood plasma (approx. pH 8).

We calculated the normalized long-range electrostatic attractions between different NPs interacting with the same cell and biological environment. The NS, ZP, and the ionic strength of the environment only affect the relative interaction energies between the NP and cell. Figure 6 shows the normalized interaction energies of NPs, predicting a negligible difference in the relative strength of NP attractions to cancerous interstitial fluid and healthy blood.

When considering the impact of the cell ZP on the long-range electrostatic interactions, negatively charged cancerous cell results in cell-ZnO NP attraction energy, while a positively charged healthy cell results in repulsive energy. Furthermore, a linear increase in the NP-Cell interaction energies is displayed for large magnitudes of cell ZPs. For example, an increase in the cell ZP magnitude from 5 mV to 35 mV strengthens the absolute interaction energy by approximately seven-fold. This trend continues for all cell ZP magnitudes analyzed in this work.

### 3.4. Association Rates

**Figure 7:**
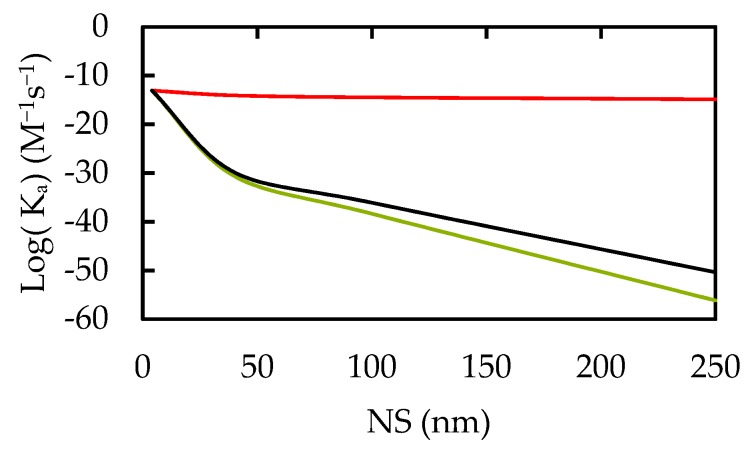
NP-Cell Association Rate Constants. Taking cells of 25 µm diameters. Red predicts the NP-cell association rate for cells of −94 mV surface potentials or pH 8 in all biological media. With cells having +5 mV and +94 mV surface potentials at pH 5, green and black respectively give this rate for NPs in interstitial and intracellular media.

Our results for the NP-Cell association are displayed in Figure 7. At high pH and small NS, the biological fluids had no influence on the association rate. However, it is increased in acidic environments where the cell potential is positive. As the NS increases from 4 to 250 nm, the difference between the NP affinities for healthy (green and black lines) and cancerous cells (red lines) becomes more pronounced in acidic, cancerous environments. Additionally, pH is predicted to be less significant for lower cellular ZPs.

**Figure 8:**
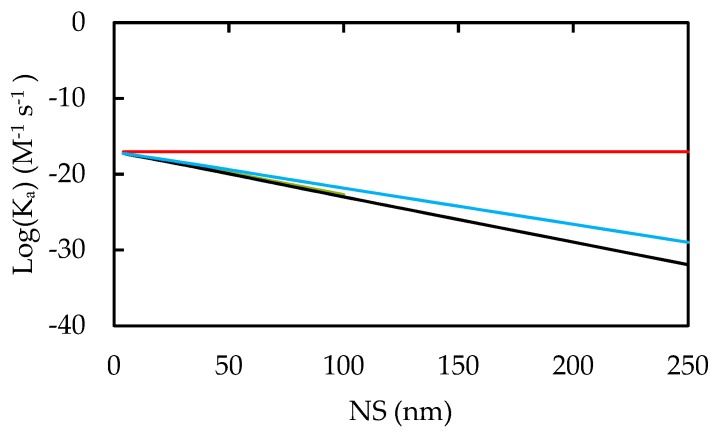
NP-NP Association rate constants. Predictions are given for pH 8 in all biological media in red, and for blood (green; collinear to black), interstitial fluid (black), and intracellular (blue) fluids at pH 5.

Our results for NP agglomeration and colloidal stability are provided in Figure 8. Near the IEP and for small NS, NP agglomeration rates are maximized. Larger NPs were found to exhibit more variance between the biological fluids in their NP-NP rather than Cell-NP interactions, with interstitial and intracellular fluids being the smallest and the largest association rates, respectively.

### 3.5. Zinc and Oxygen Free Ions Released into the Cytoplasm

The excess ion counts for a NP is necessarily dependent upon the bulk density of ions in the environment. Rather than considering the ion dissolution from the NP into the environment, we model the ions trapped or excluded from the vicinity of a NP to result in effective increases in environmental conditions.

**Figure 9:**
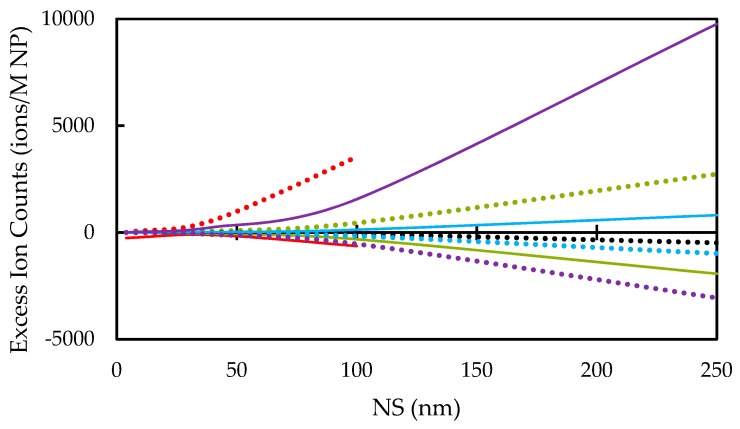
Intracellular excess zinc and oxygen ion counts (ions/Molar). Dotted lines predict excess oxygen ion counts and solid lines predict excess zinc ion counts at pH 6 (red), 7 (green), 8 (black), 9 (blue), and 10 (purple). A positive excess indicates ions trapped in the double layer.

Our results on the excess ion counts for zinc and oxygen ions in intracellular fluids are provided in Figure 9. Excess zinc ion counts are negative at pH below the IEP due to the overall positive ZP of the NP, repulsing them from the electrical double layer. When the NP is highly charged at extreme pH, the most ions are trapped or excluded in the environment.

As NS increases from 4 nm to 250 nm, the ionic release increases, though, the excess ion count per ions in the NP decreases. For example, a NS of 250 nm exhibits an excess ion count of three orders of magnitude higher than a NS of 4 nm; however, the latter NS shows an excess ion count per ions two orders of magnitude greater than that former.

## 4. Discussion

### 4.1. General Consideration

NP electrostatic properties are only dependent on NS for small NP’s in which the curvature affects the ion packing. For NSs greater than 40 nm, the ZP and SCD reach the limiting flat surface values and, consequently, the electrostatic properties of the NP do not depend on the NS anymore. Under these conditions, differences in NP behavior arise from biological considerations such as the biological filtration of NPs [15], involving hydrodynamic properties. This phenomenon explains why fenestrated or porous tissues filter larger NPs resulting in more significant NP concentrations found in many liver and kidney samples after NP treatment [15,23].

Our results for the neutralization of NP charge reveal an IEP value approximately equal to 8.2. While it is lower than the pH 9 to 10 predicted by many sources [2,44,45,46,47], it is within the ranges estimated by other sources [48,49]. Our IEP prediction is still high enough to generate positive SCDs and ZPs at the normal biological pH of 7.4, with minimal dependence on biological media [41]. Thus, ZnO NPs are well suited for attractive electrostatic interactions with negatively charged cancerous cells [2]. In the following sections, we discuss our results in conjunction with the findings of previous work to discuss possible optimization mechanisms towards the potential application of ZnO NPs for innovative cancer therapies.

### 4.2. Blood Circulation Time

Upon entry into the bloodstream at the typical biological pH of 7.4 [41], ZnO NPs with NS between 5 nm and 250 nm would experience low ZP between 3 and 8 mV. These values are expected to prevent significant aggregation, with larger NPs experiencing the least.

Previous work [16] suggests that an optimal NS of 100 nm in diameter, slightly greater than the pore size of the tumorous tissue, would be able to enter the tissue but become trapped inside. This NS would also target the liver, kidneys, and spleen to a high degree, which is mostly phagocytic [15]. Thus, any build-up of NPs in these tissues may result in aggregation [50], followed by phagocytosis [7,15], damaging healthy tissues. To avoid physical filtering through these fenestrated tissues, the NS should be less than about 50 nm in diameter [15,17,23]. On the other hand, the NS must be larger than 5.5 nm in diameter to avoid renal clearance. Our results for NP-NP association rates show faster aggregation for small NSs. Additionally, the ZP for large NS is so small in blood plasma that ZnO NPs may more easily avoid rapid bodily clearance into the reticuloendothelial system through electrostatic means [2,14,51]. Consequently, the larger end of the desirable NS spectrum would be best for the prevention of high-level aggregation and circulation for longer periods.

Another relevant parameter affecting the NP circulation time is the effective diffusion D_eff_ through the microvasculature. It relates the longitudinal transport and the NP’s molecular diffusion D_m_ resulting in axial dispersion. The ratio D_eff_/D_m_ provides an estimate for a NP’s tendency to remain in the microcirculatory flow [18]. Our results show that large NPs are more driven by microcirculatory flows caused by a decrease in the molecular diffusivity, which, in turn, increases the Peclet number relating convection to diffusion [1,18]. Thus, smaller NPs are more exposed to microvessel walls and face greater tissue uptake, which is in agreement with experiments. We also find that the blood rheological parameter ξ increases as microvessels increase in size. This increases the effective permeability parameter of the walls and drives convection, resulting in NPs being drawn away from smaller capillaries. Therefore, the lifetime of NPs in larger capillaries, arterioles, and venules [18] increase as NPs avoid being filtered out by non-target tissues. Conversely, smaller NSs may more rapidly infiltrate the micro-vasculature of the body. This result agrees with previous work [2,6,52,53], which suggests that decreasing NS results in increasing cytotoxicity. Higher NP concentrations would accumulate NPs in micro-vessels, which increase NP exposure to the interior tissues of bodily organs and potential cytotoxicity.

The analysis mentioned above on NP circulation time in terms of effective NP diffusion simplifies to only consider capillary size. Since the low hydraulic conductivity of microvessels [18,42] generates a negligible difference in the behavior of the NPs in skeletal, tumorous, and kidney capillaries, there is not enough transport of fluid through vessel membranes to significantly contribute to the dispersion of NPs toward the walls. Consequently, this model is incapable of distinguishing between healthy and tumorous micro-vasculature. In the next section, we discuss other fundamental property with which smart NPs may target tumors, namely their long-range electrostatic interaction when they enter capillaries and come close enough to bring this targeting to bear.

### 4.3. Long-Range Targeting

After the NP circulates through the body, it must be attracted to cancerous tissues and become enveloped in the cancerous interstitial fluid. Even after a NP finds itself in this interstitial medium, there must again be an attractive force to draw the NP out of the bulk extracellular environment towards the cancerous cells themselves.

While the positively or negatively charged nature of blood vessels and red blood cells differs from location to location within the body [54,55,56,57], we examined the behavior of NPs through both positively and negatively charged micro-vessels. Positive ZnO NPs would experience repulsive forces, which may drive them away from the walls and into their interior of healthy micro-vessels. This repulsion will result in prolongation of the circulation time and prevention in the uptake into positively charged vessels. On the other hand, our results on negatively charged micro-vessels (e.g., tumorous tissues [2] and some healthy tissues [54,55,56]) indicate that NPs with NS 250 nm near 12.5 µm radius cells of −38 mV would experience near-zero interactions beyond a distance equivalent to the cell’s radius. Thus, negatively charged vessels may electrostatically induce total NP clearance in micro-vessels smaller than 25 µm. As these smaller vessels have also been shown to experience reduced probabilities of containing NPs [18], this effect may be slowed over more extended periods in the case of a systemic dose.

Microvascular fenestrations and discontinuities present in cancerous capillaries provide a mean for NPs to enter the tissue [1]. High interstitial fluid pressures found in the interstitium arise from the lack of a functioning lymphatic system. This pressure generates an outward convection on the cancerous tissue fringes, driving NPs away from the interior of the tissue [1,42]. Thus, strong attractive, long-range electrostatic interactions are needed to pull positively charged ZnO NPs across the contrary convective gradient and penetrate the interior of the cancerous tissue. The opposite effect is expected to occur with healthy cells. They would repel ZnO NPs due to their positively charged nature, preventing them from becoming targets of the NP cytotoxicity. Our quantitative analysis on NS effects in these long-range interactions indicates that a mere NS increase from 16 to 24 nm increases the attractive force by about 3.5 orders of magnitude. Whereas, the viscous drag, related to the NP’s tendency to be swept away by convection, increase by 50% only (Stokes’ Law). The maximization of a NP’s long-range electrostatic affinity for a cancerous cell is thus only limited by the optimal blood circulation and micro-vessel size. Larger NSs will be exponentially more attracted to a cancerous cell, but they will also be subject to greater micro-vessel constraints. Once the NP reaches the cancerous tissues, the NP must be taken up by a cell to result in cytotoxicity. This process consists of two steps: binding (or association) and internalization, which are discussed below [17].

### 4.4. Short-Range Targeting

During the binding step, the pH of the interstitial fluid in tumor tissues is much lower than the normal blood pH of the body [58]. In acidic environments, NPs exhibit strong electrostatic attractions toward negatively charged (cancerous) cells and repulsive attractions toward positive (healthy) cells and other NPs. As NS gets larger, the repulsive forces strengthen due to the increased charge present in NPs. Near the IEP and for oppositely charged particles, the attractive forces are dominated by intrinsic London dispersion since the ZP becomes too small to drive electrostatic attraction.

The ratio of the association rates of NPs between cancerous and healthy cells is used in this work to investigate the preferential cytotoxicity of ZnO NPs towards cancerous cells. For example, a 40 nm NP would target a cancerous cell of −94 mV four orders of magnitude more than a healthy cell. The increase of NS tends to raise the responsiveness of the cancer-healthy cell ratio. Consequently, more negatively charged cancerous cells, found at lower pH environments [2], would result in higher association rates for larger NSs.

A similar analysis predicts that larger NPs tend to aggregate less than smaller NPs. Additionally, the NP aggregate would have smaller overall volume when comprised of larger NPs, aiding in circulation and transport. Since the intracellular fluid is more ionic than interstitial fluid, it decreases the ZP of the NP and weakens the electrostatic repulsion between particles. This allows NP-NP and NP-cell association more readily in intracellular fluid.

We conclude that NPs on the larger end of the desirable NS spectrum would provide the most suitable smart-NP properties for avoiding rapid bodily clearance and selectively targeting cancerous cells.

### 4.5. NP Internalization and Cytotoxicity

It is well-known that internalization may occur through phagocytosis, endocytosis, or other less significant means [2,7,15,16,20,21,22,59]. While endocytosis holds the most promising approach for singular NPs to enter a cancerous cell, phagocytosis accounts for NP aggregates that may form.

Receptor-mediated endocytosis involves the intake of relatively small amounts of material into the cell via the triggering of specific receptors on the surface of the cell. This process would require the NP surface to be functionalized in a manner that may affect its electrostatic characteristics, which is beyond the scope of this research. However, endocytosis may still occur, even without the use of receptors, namely, when the NP-cell interface is energetic enough. [7,20,21,22,59]. Previous works are based on a purely kinetic approach that does not consider the NP material but its size. It was found that endocytosis is strongly size-dependent, as opposed to charge-dependent, with the optimal size predicted approximately 30 nm in diameter [7,21,22,59]. It was also found that larger NPs need less energy at the NP-cell interface to endocytose, while more energetic NPs need to be smaller for optimal uptake. This result fits well within the desirable NS range between 5.5 nm and 50 nm in diameter.

Based on this analysis, we find optimal conditions for NPs of 20 nm in diameter with ZP of as much as 20 mV and a cancerous cell ZP of as much as −94 mV [31]. This configuration would provide a favorable energetic NP-cell interface and may decrease the optimal uptake NS as well. On the other hand, NP associated to the surface of a healthy cell would face a relatively unenergetic NP-cell interface due to the positive charge nature of the health cell. Consequently, we would expect this NS to endocytose readily with a cancerous cell and add another layer of preferential cytotoxicity towards cancerous cells.

Phagocytosis is however the primary method by which NPs may enter the cell. This internationalization process involves the consumption of much larger quantities of matter by the cell. In this situation, NPs are brought by receptors triggered by protein coronas that are often formed around charged NPs [7,23]. Protein coronas have also been found to influence the rate of uptake only, no matter whether the cell internalizes the NP [15]. Previous work suggests optimal NSs be greater than 500 nm in diameter [7], whereas it should approximately be 50 nm according to others [15]. According to Heng [53], particles of 500 nm may form from aggregates at pH near IEP but less than in more neutral pH fluids [48]. On the other hand, NS 50 nm is at the maximum end of the optimal NS range to avoid physical filtering. Nevertheless, without aggregation, it would result in NP uptake through the phagocytic pathway just as readily as the endocytic.

Once the NPs enter the cells, the final stage involves a series of mechanisms generally placing the NPs and/or agglomerates inside the cellular lysosome. Within this environment, the pH ranges between 4 and 5.5, and, consequently, NPs would begin to dissolve, releasing free zinc and oxygen ions into the environment. They would also increase their ZP well beyond the 30-mV predicted at pH 6. Under these conditions, any NP agglomerate would break down into its individual NPs [53]. The resulting cytotoxic free ions and highly charged NPs would begin to produce ROS, react with the environment, escape the lysosome, and induce apoptotic mechanisms within the cancerous cell [2,6,8,9,10].

NPs can introduce free zinc and oxygen ions to the body either through dissolution or through the exclusion/trapping of ions already present. This results in cytotoxicity. Considering that a NP can capture/exclude more ions in its double layer than it contains, the latter of these methods is highly relevant. Unlike the experimental limitations existing to directly measure ion production by NPs [6,7,8,9,10,11], the present complexation surface model introduced for ZnO NPs provides a novel way to estimate the excess ion counts afflicted on a cell (shown in Figure 8).

It is worth noting that our results on cytotoxicity in biological fluids without NP dissolution would be in contradiction with experimental results, which often show both increased levels of both ROS and zinc concentrations with decreasing pH [6,11]. These free ions may quickly bind and react with other substances to induce cytotoxicity [6,38,60]. However, with zinc and oxygen ions carrying opposite charges, our calculations show opposite signs in the excess ion counts for each of these species under most conditions. This model predicts that positive SCDs would repeal zinc ions and attract oxygen ions, and vice versa. A dissolution model would instead increase concentrations of both zinc and oxygen ions. Both models would be present in the biological environment and both need to be considered to accurately predict experimental results. We conclude that larger NSs entering the cell would cause higher cytotoxicity. This would be a result of the greater exclusion volume of the NP double layer and the greater NP charging due to its size. Our results near the IEP show a minimum magnitude in the excluded ion counts and the relative toxicity. This result indicates that both the exclusion and the dissolution models of toxicity approach a minimum near the IEP, just above healthy biological pH [4]. Toxicity due to free zinc ions would also approach a minimum with the decrease in both dissolution and excluded ion counts.

## 5. Conclusions

In this article, we combined a multiscale approach, a unique nanoparticle model, and available experimental data to characterize the behavior of zinc oxide nanoparticles under different vessels rheology, pH levels, and biological environments. We used this characterization to produce extensive, valuable, novel information on the impact of pH and biological environment alterations in the charging mechanisms of zinc oxide nanoparticles; vessel permeability and blood rheology in the nanoparticle circulation; surface charge density and nanoparticle size in nanoparticle aggregation, NP-cell association rates, selectivity, and toxicity. Our predictions are in good agreement with previous work, mostly experimental data. In such situations, we were not only able to validate and support our models and theories, but also to identify and characterize the molecular mechanisms governing the behavior of the nanoparticles. We also provided new findings where experimental data are still unavailable. This was possible by considering an extensive, yet unexplored, range of nanoparticle sizes and pH levels, as well as, by performing novel calculations on the behavior of ZnO NP at every stage on its way to the interior of a cancerous cell. We summarize our findings below:

Nanoparticle behavior under different biological environments and nanoparticle sizes

- Geometrical considerations reveal that the electrostatic properties of the nanoparticle do not depend on the nanoparticle size for radii greater than 40 nm. In such a situation, the nanoparticle behavior is governed by their hydrodynamic properties.
- The nanoparticle charge is fully neutralized at a pH value of approximately equal to 8.2, more significant than the physiological and pathological values.
- Significantly lower values for zeta potential and surface charge density are present in intracellular fluids compared to those obtained for interstitial/blood plasma.

Nanoparticle circulatory transport

- Considerations of blood rheology and vessel permeation reveal a substantial increase in zinc oxide nanoparticle diffusivity with increasing capillarity and nanoparticle size. Thus, nanoparticle sizes injected into large vessels of the bloodstream are subject to higher diffusion than into small vessels. Small nanoparticle sizes may more rapidly infiltrate the micro-vasculature of the body. Whereas, large nanoparticle sizes increase the blood circulation time with decreased nanoparticle transfer into micro-vasculature of the body.
- Low hydraulic conductivities found in tissues generate a negligible difference in the behavior of the nanoparticles in skeletal, tumorous, and kidney capillaries.

Long-Range interaction (selectivity process)

- Long-range electrostatic interactions may interfere with the effective diffusivity of nanoparticles in the micro-vasculature. Positively charged vessels may limit uptake into tissues, while negatively charged vessels can result in a clearance inversely proportional to the square of the micro-vessel size.
- A negatively charged cancerous cell results in a positive cell-zinc oxide nanoparticle attraction energy, while a positively charged healthy cell results in repulsive energy. Large nanoparticle sizes are exponentially more attracted to a cancerous cell, but they are also subject to greater micro-vessel constraints.

Short-Range interactions (binding process)

- Considerations on nanoparticle-Cell association rates reveal a significant increase in the selectivity for cancerous cells versus healthy cells at large nanoparticle size and low pH.
- Large nanoparticle sizes prevent high level nanoparticle aggregation.

Nanoparticle Internalization and cytotoxicity

- Large nanoparticles need less energy at the nanoparticle-cell interface to endocytose, while more energetic nanoparticles need to be smaller for optimal uptake.
- Inside the cellular lysosome, nanoparticles dissolve, releasing free zinc and oxygen ions producing reactive-oxygen-species, reacting with the environment, escaping the lysosome, and inducing apoptotic mechanisms within the cancerous cell.

Based on these results, we conclude that spherical zinc oxide nanoparticles with surface density *N* = 5.89 × 10^−6^ mol/m^2^, protonation and deprotonation rates *pKa* = 10.9 and *pKb* = −5.5, and nanoparticle size in the range of 20–50 nm in diameter, are the most effective, smart anti-cancer agents for biomedical treatments on a systemic way. While these nanoparticles are advantageous in the great majority of situations, our approach also finds that negatively charged healthy tissues, such as osteoblasts [2,59,61], would become subject to the same cytotoxic targeting as cancerous cells.

Overall, our results provide a deeper understanding on the extraordinary capability of zinc oxide nanoparticles in adapting their physicochemical properties to its environment, remaining in circulation for prolonged periods, and actively targeting, approaching, associating with, and killing cancerous cells. Further research includes the effects of protein corona, nanoparticle functionalization, and explicit ion dissolution mechanisms on the nanoparticle complexation surface model. It is also essential to consider the impact of explicit water molecules, and zeta potential of blood vessels in the nanoparticle circulation model.

## Figures and Tables

**Table 1:** Simplified biological electrolyte compositions (mM) [41]. Note: Oxygen is used in substitution for amino acids and charged proteins to maintain electroneutrality.

Ionic Species	Blood Plasma	Interstitial Fluid	Intracellular Fluid
Sodium	151	142	11
Potassium	10	9	141
Calcium	0	2	0
Magnesium	0	1	19
Chlorine	110	118	0
Carbonate	14	30	12
Hydrogen phosphate	0	2	47
Sulfate	0	0	10
Oxygen	18.5	0.25	32

**Table 2:** Average dimensions and velocities for blood vessels.

Vessel	L (mm)	Re (mm)	U (mm/s)
Arteriole	1.5–2	0.02–0.1	5
Capillary	0.5	0.005–0.01	0.1–1
Venules	1	0.02–0.05	0.5

**Table 3:** Hydraulic conductivity and permeability parameters for capillaries of 100 micrometers length.

Organ	*L_p_* × 10^−8^ (µm/s/Pa)	II (*R_e_* = 10 µm/*R_e_* = 70 µm)
Skeletal Muscle	250	0.0010/0.00005
Tumor [42]	1500	0.0025/0.0001
Glomerulus in Kidney	15,000	0.0077/0.0042

**Table 4:** Inlet and outlet pressures for capillaries, as well as the interstitial pressure.

Organ	Inlet (mmHg)	Outlet (mmHg)	Interstitial (mmHg)
Skeletal Muscle	30	15	4.7
Tumor [42]	30	15	22
Glomerulus in Kidney	35	15	−1

## Supplementary Materials

The following are available online at https://www.mdpi.com/2079-4991/10/2/269/s1.
