# Supplementary material for: The Self-Adaptation Ability of Zinc Oxide Nanoparticles Enables Reliable Cancer Treatments
Source: Nanomaterials (Basel). 2020 Feb 5;10(2):269. doi: 10.3390/nano10020269 (PMC7075113; doi:10.3390/nano10020269)
Supplement: Supplementary file 1 [file nanomaterials-10-00269-s001.pdf]

nanomaterials

MDPI

Article

The Self-Adaptation Ability of Zinc Oxide Nanoparticles Enables Reliable Cancer Treatments

Zane Taylor [1,2] and Marcelo Marucho [2,*]

1 Department of Applied Physics and Materials Science, California Institute of Technology, Pasadena, CA 91125, USA; zwtaylor76@gmail.com

2 Department of Physics and Astronomy, University of Texas at San Antonio, One UTSA Circle, San Antonio, TX 78249, USA

* Correspondence: marcelo.marucho@utsa.edu

Received: 10 December 2019; Accepted: 3 February 2020; Published: 5 February 2020

1. Supplementary Material

1.1. Nanoparticle Model: pKa and pKb Calculation

We use the Pivovarov formula to calculate a surface site density of locations capable of protonation or deprotonation. The corresponding result is N = 5.89×10^{-6} mol/m^2, which is comparable in magnitude to the surface site density of other metal oxides such as silica oxide. [1] We notice that this large value of ZnO surface site density is in agreement with the findings of other work on material pH sensitivity [2].

Subsequently, we use the value for the parameter value N and assign different values for the parameters pKa and pKb, the protonation and deprotonation constants, respectively, into the complexation surface model introduced in reference 27 to predict values for ZnO NP ZPs (see Figure S1). We find that the fitting values pKa = 10.9 and pKb = −5.5 reproduce all available experimental results on ZnO NP ZP [3–7]. These values fall within the wide range of values found in literature, which suggest values anywhere from −5.7 to 15.7 for pKa alone [2,3,8,9].

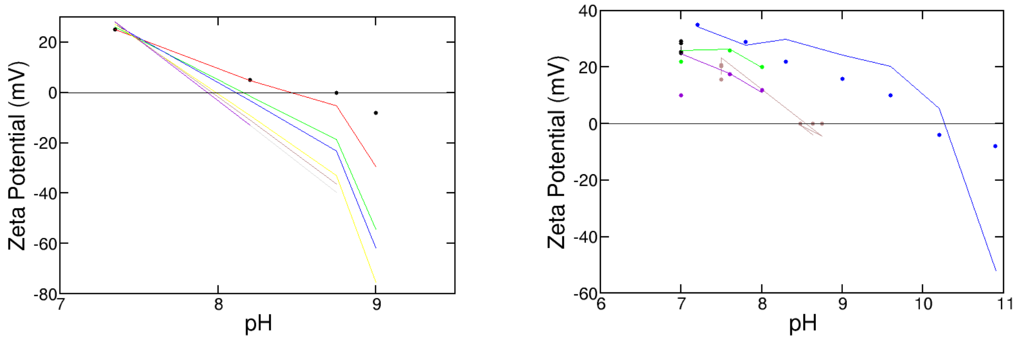

(A) (B)

Figure S1. (**A**) Comparison of the zeta potential vs pH results for different pKa and pKb pairs in NS 40A. Black circles represent experimental data [4] for ZnO NPs. The solid lines represent CSDFT predictions for a number pKa and pKb pairs, notated as (pKa,pKb): red (10.9,−5.5), green (10.2,−5.6), blue (10.0,−5.7), yellow (9.65,−5.8), brown (9.50,−5.9), grey (9.35,−6.0), and purple (9.2,−6.1). (**B**) Comparison of ZP vs pH results for different experimental conditions (circles) and CSDFT predictions

with the same experimental input (solid lines). Different colors represent different sources and experimental conditions: Black [10], Green [5], Blue [6], Brown [4] and Purple [11].

1.2. Nanoparticle Transport

We use Gentile et. al.'s expression [12] to calculate the effective diffusion of spherical particles

$$D_{eff} = D_m\left[1 + \frac{Pe_e^2}{48}\left(\frac{cosh(\bar{z}\Gamma(\xi)) - \Omega cosh(\Gamma(\xi) - \bar{z}\Gamma(\xi))}{1 - \Omega cosh(\Gamma(\xi))}\right)^2 G(\xi)\right]$$

where,

$$D_m = \frac{k_B T}{6\pi\eta R_{NS}}$$

$$P_e = \frac{R_e U}{D_m}$$

$$\Gamma(\xi) = \frac{\Pi}{\sqrt{A(\xi)}}$$

$$\eta_N = \eta_P(1 + 2.5H + 7.35H^2)$$

$$\bar{z} = \frac{z}{l}$$

with k_B, the Boltzmann constant, T, temperature, η and η_N, the blood viscosity, η_P, the blood plasma viscosity, R_{NS}, the nanoparticle radius, P_e, the Peclet number, R_e, the vessel radius, U_e, the average velocity of blood in the vessel (provided in Table 5), z, the position along the axis within the vessel, l, the total length of the vessel, H, the hematocrit level of the blood, Π, the permeability of the vessel, and

$$\Omega = \frac{p_0 - \pi_i}{p_1 - \pi_i}$$

$$\xi(R_v) = 1 - 3R_v^{-0.8}$$

$$A(\xi) = 1 - \frac{16}{7}\xi^{\frac{1}{2}} + \frac{4}{3}\xi - \frac{1}{21}\xi^4$$

$$G(\xi) = 7 - \frac{279424}{8085}\xi^{\frac{1}{2}} + \frac{3655264}{56595}\xi - \frac{5679616}{105105}\xi^{\frac{3}{2}} + \frac{256}{15}\xi^2 - \frac{208}{147}\xi^4 + \frac{3328}{1029}\xi^{\frac{9}{2}} - \frac{832}{441}\xi^5 - \frac{16}{55}\xi^6 + \frac{128}{165}\xi^{\frac{13}{2}} - \frac{32}{55}\xi^7 + \frac{22007}{200655}\xi^8 - \frac{8}{147}\xi^4(-42 + 96\xi^{\frac{1}{2}} - 56\xi + \xi^4)ln(\xi)$$

In the latter equations, R_v is the vessel radius, ξ, the rheology of the blood, π_I, the interstitial fluid pressure, and, p_o and p_1, the inlet and outlet pressures of the vessel, respectively.

In this way, the greater the effective diffusion, the smaller the circulation time of the NP in the body because it is more readily coming in contact with tissues that will remove it from the blood stream. Thus, for a longer circulation time in the body, we would desire a NP with a low effective diffusion.

1.3. Long-Range Electrostatic Interaction

Once the NP approaches the tissues themselves, the long range electrostatic interactions between the NP and the cells becomes the driving force behind NPs approaching cells and discriminating between cancerous and healthy cells. This long-range interaction is modelled by Ohshima et al.[13] using a modified Columbic force to take the effects of the electrolyte into account. The interaction energy is defined as:

$$V(r) = 4\pi\varepsilon_r\varepsilon_0\left(\frac{k_BT}{q}\right)^2 r_1r_2\bar{Y}_1\bar{Y}_2\frac{1}{r}exp[-\kappa(r - r_1 + r_2)]$$

where,

$$\bar{Y}_i = 8tanh\left(\frac{y_{s_i}}{4}\right)\left[\frac{1}{1+\left\{1-\frac{2A_i+1}{A_i+1^2}tanh^2\left(\frac{y_{s_i}}{4}\right)\right\}^{\frac{1}{2}}}\right]$$

$$y_{s_i} = \frac{q\psi_{s_i}}{k_BT}$$

$$A_i = \kappa r_i$$

and all other parameters were previously defined in this appendix: q is the charge of an electron, ε_r and ε_0, the relative permittivity of the substance (water) and free space respectively, r_1 and r_2, the radii of the two particles involved in the interaction, r, the separation distance of the particles, κ, the inverse Debye length of the fluid, and ψ_s the surface potential of the particle.

When considering $V(r)$ in a normalized form, $V_n(r)$, we obtain a simpler (dimensionless) expression which depends only upon the NP and electrolyte characteristics, with no relationship to the separation distance between the NP and cell, or to the cell itself. This expression reads

$$V_n(r) = \frac{r_1}{r_N}\frac{\bar{Y}_1}{\bar{Y}_N}e^{\kappa(r_1 - r_N)}$$

where the subscript N represents the baseline NP upon which the normalization is to occur, namely, a ZnO NP of 4 nm in radius.

1.4. DLVO Theory

Trefalt and Borkovec [14] provide a method, based on the DLVO theory, for calculating the association rate k

$$k = \frac{2k_BT}{3\eta R_{eff}}\left\{\int_0^\infty \frac{B(h)}{(R_1+R_2+h)^2}exp\left[\frac{U(h)}{k_BT}\right]dh\right\}^{-1}$$

$$B(h) = 1 + \frac{R_{eff}}{h}$$

$$R_{eff} = \frac{R_1R_2}{R_1+R_2}$$

given a function of the potential energy of attraction $U(h)$ between the two particles as a function of center-to-center separate ion distance h.

Ohshima[15] introduces a potential model which is appropriate for materials that react with their environments to exchange surface groups and modulate their surface charge, especially as they

approach one another and those surface group's ability to be exchanged becomes inhibited [16]. The approach provides the following expression for the potential energy function

$$U(h) = 4\pi R_{eff}\varepsilon_r\varepsilon_0\psi_1\psi_2 e^{-\kappa h} - \frac{H}{6}(C_1 + C_2 + C_3)^2$$

$$\kappa^{-1} = \left(\frac{k_B T\varepsilon_r\varepsilon_0}{2q^2 N_A I}\right)^2$$

$$I = 1000\frac{1}{2}\sum_i z_i^2 c_i$$

with N_A being Avogadro's number and

$$C_1 = \frac{2R_1R_2}{(h + R_1 + R_2)^2 - (R_1 + R_2)^2}$$

$$C_2 = \frac{2R_1R_2}{(h + R_1 + R_2)^2 - (R_1 - R_2)^2}$$

$$C_3 = ln\left\{\frac{(h + R_1 + R_2)^2 - (R_1 + R_2)^2}{(h + R_1 + R_2)^2 - (R_1 - R_2)^2}\right\}$$

The Hamaker constant $H = H_{132}$ for the NP-NP and NP-Cell interactions may be approximated if some basic information for each medium is known [15,17,18]. e.g.

$$H_{132} = (\sqrt{A_1} - \sqrt{A_3})(\sqrt{A_2} - \sqrt{A_3})$$

where $A_1 = A_{\text{ZnO-vacuum-ZnO}} = 9.21 \times 10^{-21}$ J, $A_2 = A_{\text{phospholipid-vacuum-phospholipid}} = 8 \times 10^{-21}$ J, and $A_3 = A_{\text{water-vacuum-water}} = 55 \times 10^{-21}$ J. With these values, the approximated Hamaker constant for NP-NP interactions is 2.01×10^{-20} J and that for NP-Cell interactions is 1.92×10^{-20} J.

The cell membrane potential ψ_2 is provided by several sources [19,20]. These sources note that cancerous cells generally have more negative potentials than healthy cells, however, there are many negatively charged healthy cells. For example, healthy skeletal muscles exhibit a potential of as low as −94 mV. On the other hand, healthy cells may additionally reach membrane potential values of nearly 60 mV, as in the case of normal fibroblasts. Thus, we let these extreme negative values represent a minimum potential for cancerous cells, we additionally let a value of +94 mV represent the absolute maximum potential of healthy cells.

1.5. Ion Count Calculations

Using normalized density profiles $\rho(r)$ consisting of the local molarity of an ion divided by the bulk molarity of the electrolyte, as a function of distance from the center of the NP, the total excess ion counts *eic* may be calculated [21]. Integrating over the domain of these figures, we get

$$eic = \int 4\pi r^2(\rho(r) - 1)dr$$

With this equation, we receive a unit of molÅ^3/L. To make this into the excess number of ions per molar of bulk electrolyte trapped by the EDL, we multiply it by the Avogadro's number and divide by a factor of 10^{27}.

1.6. Stokes' Law & Bernoulli's Equation

Stokes' Law relates the velocity of a spherical particle in a fluid v (or the velocity of the fluid around the particle) to the drag force acting on that particle F_D. Using this approach, the drag force a NP would experience in a convective current and, thus, the force required to pull the NP against such a current, can be approximated by

$$F_D = 6\pi\eta Rv$$

where η represents the viscosity of the fluid, R, the NS, and v the relative velocity.

The fluid velocity may further be approximated using the Bernoulli expression

$$P_1 + \frac{1}{2}\rho v_1^2 + \rho g h_1 = P_2 + \frac{1}{2}\rho v_2^2 + \rho g h_2$$

modified to consider only a change in pressure versus a change in velocity at constant elevation and zero initial relative velocity.

$$\frac{2\Delta P}{\rho} = (\Delta v)^2$$

References

1. Hunley, C.; Marucho, M. Electrical double layer properties of spherical oxide nanoparticles. *Phys. Chem. Chem. Phys.* **2017**, *19*, 5396–5404.
2. Al-Hilli, M.S.; Al-Mofarji, R.T.; Willander, M. Zinc oxide nanorod for intracellular pH sensing. *Appl. Phys. Lett.* 2006, **89**.
3. Dirkse, T.P. The Solubility Product Constant of Zno. *J. Electrochem. Soc.* **1986**, *133*, 1656–1657.
4. Mudunkotuwa IA, Rupasinghe T, Wu CM, Grassian VH. *Dissolution and Aggregation of Zinc Oxide Nanoparticles at Circumneutral pH: A Study of Size Effects in the Presence and Absence of Citric Acid*; Langmuir : the ACS Journal of Surfaces and Colloids: Washington, DC, USA; 2012; 396–403 p.
5. Omar, F.M.A.; Stoll, S. Stability of ZnO Nanoparticles in Solution. Influence of pH, Dissolution, Aggregation and Disaggregation Effects. *J. Colloid Sci. Biotechnol.* **2014**, *3*, 10.
6. Marsalek, R. Particle size and Zeta Potential of ZnO. *APCBEE Procedia* **2014**, *9*, 5.
7. Sayes, C.M.; Berg, J.M.; Romoser, A.; Banerjee, N.; Zebda, R. The relationship between pH and zeta potential of similar to 30 nm metal oxide nanoparticle suspensions relevant to in vitro toxicological evaluations. *Nanotoxicology* **2009**, *3*, 276–283.
8. Lim, E.; Pon, A.; Djoumbou, Y.; Knox, C.; Shrivastava, S.; Guo, A.C.; Neveu, V.; Wishart D.S.; T3DB: a comprehensively annotated database of common toxins and their targets. *Nucleic Acids Res.* **2010**, 38, 781–786.
9. Available online: http://foodb.ca (access on 7 February 2020)
10. Yu, J.B.; Chung, H.E.; Choi, S.J. Effects of Physicochemical Properties of Zinc Oxide Nanoparticles on Cellular Uptake. *J. Phys. Conf. Ser.* **2011**, *304*, 012007.
11. Degen, A.; Kosec, M. Effect of pH and impurities on the surface charge of zinc oxide in aqueous solution. *J. Eur. Ceram. Soc.* **2000**, *20*, 667–673.
12. Gentile, F.; Ferrari, M.; Decuzzi, P. The transport of nanoparticles in blood vessels: the effect of vessel permeability and blood rheology. *Ann. Biomed. Eng.* **2008**, *36*, 254–261.
13. Ohshima, H.; Healy, T.W.; White, L.R. Accurate Analytic Expressions for the Surface-Charge Density Surface-Potential Relationship and Double-Layer Potential Distribution for a Spherical Colloidal Particle. *J. Colloid Interface Sci.* **1982**, *90*, 17–26.
14. Trefalt, G.; Ruiz-Cabello, F.J.M.; Borkovec, M. Interaction Forces, Heteroaggregation, and Deposition Involving Charged Colloidal Particles. *J. Phys. Chem. B* **2014**, *118*, 6346–6355.
15. Ohshima, H. *Electrical Phenomena at Interfaces and Biointerfaces: Fundamentals and Applications in Nano-, Bio-, and Environmental Sciences*; Wiley: Hoboken, NJ, USA, 2012; 850 p.
16. Mikelonis, M.A.; Youn, S.; Lawler, D.F. DLVO Approximation Methods for Predicting the Attachment of Silver Nanoparticles to Ceramic Membranes. *Langmuir* **2016**, *32*, 1723–1731.

17. Bergstrom, L. Hamaker constants of inorganic materials. *Adv. Colloid Interface Sci.* **1997**, 70, 125–169.
18. Leite, F.L.; Bueno C.C.; DaRóz A.L.; Ziemath E.C.; Oliveira O.N. Theoretical Models for Surface Forces and Adhesion and Their Measurement Using Atomic Force Microscopy. *Int. J. Mol. Sci.* **2012**, 13, 12773–12856.
19. Yang, M.; Brackenbury, W.J. Membrane potential and cancer progression. *Front. Physiol.* 2013, **4**.
20. Binggeli, R.; Cameron, I.L. Cellular-Potentials of Normal and Cancerous Fibroblasts and Hepatocytes. *Cancer Res.* **1980**, *40*, 1830–1835.
21. Ovanesyan, Z.; Medasani, B.; Fenley, M.O.; Guerrero-García, G.I.; Olvera de la Cruz, M.; Marucho, M. Excluded volume and ion-ion correlation effects on the ionic atmosphere around B-DNA: Theory, simulations, and experiments. *J. Chem. Phys.* 2014, **141**.

© 2020 by the authors. Submitted for possible open access publication under the terms and conditions of the Creative Commons Attribution (CC BY) license (http://creativecommons.org/licenses/by/4.0/).
